# Sun, sea and sex: a review of the sex tourism literature

**DOI:** 10.1186/s40794-020-00124-0

**Published:** 2020-11-27

**Authors:** Timothy Siliang Lu, Andrea Holmes, Chris Noone, Gerard Thomas Flaherty

**Affiliations:** 1 School of Medicine, National University of Ireland Galway, Galway, Ireland; 2 Saolta University Hospital Healthcare Group, University Hospital Galway, Galway, Ireland; 3 School of Psychology, National University of Ireland Galway, Galway, Ireland; 4 School of Medicine, International Medical University, Kuala Lumpur, Malaysia

**Keywords:** Sex tourism, Commercial sex work, Sexually transmitted infections, STI, Human immunodeficiency virus, HIV, Sexual violence

## Abstract

**Background:**

Sex tourism is defined as travel planned specifically for the purpose of sex, generally to a country where prostitution is legal. While much of the literature on sex tourism relates to the commercial sex worker industry, sex tourism also finds expression in non-transactional sexual encounters. This narrative review explores current concepts related to travel and sex, with a focus on trans-national sex tourism.

**Methods:**

The PubMed database was accessed to source relevant literature, using combinations of pertinent search terms. Only articles published in the English language were selected. Reference lists of published articles were also examined for relevant articles.

**Results:**

With regard to preferred destinations, South/Central America and the Caribbean were more likely to receive tourists looking for casual sex. Longer duration of travel, travelling alone or with friends, alcohol or drug use, being younger and being single were factors associated with higher levels of casual sex overseas. The majority of literature retrieved on sex workers focused on risk behaviours, sexually transmitted infections (STI), mobility of sex workers and how these factors affected their lives. Sex tourists require better access to effective methods of preventing HIV, such as pre-exposure prophylaxis, and better education on HIV prevention. Drugs and alcohol play a major role as risk factors for and cofactors in casual sexual behaviour while abroad.

**Conclusions:**

Travellers need to be informed of the increased risks of STI before travel. They should be aware of the local prevalence of STIs and the risks associated with their sexual practices when they travel, including engaging with commercial sex workers, having unprotected sexual intercourse and becoming victims of sexual violence.

## Background

Prior to the current pandemic of COVID-19, international travel had reached record levels of activity, with 1.4 billion traveller arrivals recorded in 2018 [1]. Sex and travel have a long association, dating from the ancient world onwards [2], and their connection is still apparent today. Sex tourism is defined by the Centre for Disease Control and Prevention (CDC) as “travel planned specifically for the purpose of sex, generally to a country where prostitution is legal” [3]. Domestic sex tourism implies travel within the same country, while trans-national sex tourism refers to travel across international boundaries.

While much of the literature on sex tourism relates to the commercial sex worker industry, which remains illegal in many jurisdictions, sex tourism also finds expression in non-transactional sexual encounters, typically involving a tourist from an economically developed country seeking sexual experiences in developing host destinations. In some cases, travellers may engage in sex tourism to validate their own sexual identity with greater freedom than would be allowed in their own, more conservative nations. The main source of opposition to sex tourism concerns the troubling phenomenon of child sex tourism, which will be explored later in this review.

The link between travelling and the spread of disease is undeniable, as demonstrated by the current COVID-19 pandemic. As the travel landscape changes in the aftermath of the pandemic, so will the behaviour of travellers. The subject of sex tourism has been neglected to date in the travel medicine literature and receives little attention in the pre-travel health consultation. This narrative review explores current concepts related to travel and sex, with a focus on trans-national sex tourism, while also giving an insight into specific risks and behaviours associated with this activity.

## Methods

### Literature search strategy

The PubMed database was accessed between June 2019 and June 2020 to source relevant literature using combinations of the following search terms: Sex, Tourism, Travel, Migration, Holiday, Abroad, Vacation, Sexually Transmitted Infection, Sexually Transmitted Disease, Human Immunodeficiency Virus, Prostitution, Drugs, Alcohol, Trafficking, Rape, Child, Military, Navy, Defence Forces, Business, Homosexual, Heterosexual, LGBTQ+, Transgender, Asia, North America, South America, Europe, Oceania, Africa. Only articles published in the English language were selected. Articles published within the past 5 years were prioritised. Reference lists of published articles were examined to ensure all relevant articles were included. Relevant sources of grey literature were also retrieved using Google® as a search engine. The legality of prostitution in different international jurisdictions, governmental attempts to regulate the sex tourism industry and the extraterritorial criminalisation of child sex tourism were beyond the scope of the current review.

## Results

### Epidemiology of sex and travel

In the context of this review, we define casual sex as sexual relations undertaken without serious intent or emotional commitment between individuals who are not established sexual partners or do not know each other well. Men were more likely to seek out or engage in casual or risky sex behaviours (e.g., multiple partners, unprotected intercourse) while travelling [4–6]. As many as 1 in 10 men were recorded as having an overseas partner in a British study [6], and different categories of male sex tourist have also been proposed in the literature [7], ranging from the ‘macho lad’ asserting his dominance over foreign women to the ‘white knight’ saving women from commercial sex work. A study from the United States showed that female travellers had a greater preference for travel to European or tropical countries, and that sex was more likely to occur on group tours, sightseeing or backpacking holidays lasting fewer than 14 days [8]. Female sex tourism has also been described in Caribbean destinations such as Jamaica, with Euro-American women purchasing the services of so-called “Rent-A-Dreads”, local men who seek out relationships with tourist women for economic gain [9]. Younger women were reported to prefer expatriates and other tourists as sexual partners [10], while men of all ages and older women were reported to exercise a preference for local partners.

With regards to preferred destinations, a meta-analysis conducted in 2018 showed that South/Central America and the Caribbean were more likely to receive tourists looking for casual sex [5]. Additionally, Thailand and Cuba also have a prevalent sex tourism industry [7, 11]. One study found that 66% of Australian tourists to Thailand were planning on having a sexual encounter while there [11], while sex tourism in Cuba has been described as “integral to the Cuban experience” [12]. Traveller subtypes who were more likely to engage in sex included backpackers, travelling businessmen, those visiting friends and relatives (VFR), and those travelling specifically to solicit commercial sex workers [5]. Factors associated with popular sex tourism destinations are described in Table 1.

**Table 1:** Characteristics of popular sex tourism destinations [5, 13–15]

Destination	Associated Characteristics
South/Central America	Greater chance of casual sex Less consistent use of contraception
Caribbean	Greater chance of casual sex Less consistent use of contraception
Western Europe	Greater risk of STI
Southeast Asia	Greater risk of STI

*STI* sexually transmitted infection

Several studies report that longer duration of travel (greater than 1 month), travelling alone or with friends, alcohol or drug use, being younger and being single were factors associated with higher levels of casual sex overseas [4–6]. A study conducted in Sweden reported conflicting data, showing that short term travellers (less than 5 days) were 20 times more likely to engage in casual sex [16]. While few studies offered information linking different ethnicities to sexual behaviour overseas, one British study found that non-white citizens were more likely to engage in sexual behaviour while travelling [6]. Migrants and members of the lesbian, gay, bisexual, transgender and queer (LGBTQ+) community are also discussed frequently in the sex tourism literature. A summary of the characteristics associated with sexual risk behaviour is shown in Table 2. This will be explored further in this review. Studies of travellers engaging in sex with tourism representatives [11], sex workers and fellow travellers [5] show that choice of partner while travelling is not limited to any particular demographic.

**Table 2:** Summary of the characteristics of a typical sex tourist [4–6, 8, 16–23]

Male	
Younger age group	
Single	
Alcohol and drug use	
LGBTQ+	
Migrant	
Travel for < 14 days	
Travel for > 1 month	

*LGBTQ+* Lesbian, Gay, Bisexual, Transgender and Queer

### Commercial sex work and travel

Travellers may engage in planned or opportunistic interactions with commercial sex workers (CSW). The majority of literature we retrieved on sex workers focused on risk behaviours, sexually transmitted infections (STI), mobility of sex workers and how these factors affected their lives. De et al. examined the different categories of sex worker in the region of Bangui in the Central African Republic, and found that 1 in 4 of ‘Pupulenge’, the higher class sex worker more likely to cater to foreigners, had poor regular usage of condoms in the previous 3 months, but better knowledge of their HIV/AIDS risk and status [24]. There were similar findings among male sex workers in Jamaica, who regarded themselves more as long term romantic partners of female tourists, and as such had low levels of condom usage [13]. In addition, these men had reported misuse of alcohol and drugs, and were accustomed to having multiple partners.

Safe sex behaviours were also shown to be highly dependent on the travel destination. A study in Singapore showed that 87.5% of local men used condoms when engaging a sex worker in Singapore, but when travelling the rate dropped to between 44 and 77%, depending on location [25]. This finding was supported by research from Hong Kong, which also showed that heterosexual men reported lower levels of condom usage when visiting sex workers outside of their own country [26]. Hsieh et al. [27] proposed that the clients of sex workers could facilitate the spread of STIs between different nations and networks to a larger degree than sex workers, while also contributing to STI prevalence within their own communities.

An interesting area with limited research evidence is the role sex tourism websites play, with only one paper identified on this subject [28]. This article analysed various sex tourism websites and found that most displayed sex workers as commodities, to be chosen and paid for by tourists, portraying them as exotic third world women, capable of providing a “total girlfriend experience”, enjoying the company of foreigners and being completely subservient to them. This study proposed that these websites enforce the fiction behind sex tourism and, in doing so, sustain the possible misogynistic views of the sex tourist. It was also noted that any legal or health information on these websites was centred round the tourist, rather than the sex worker.

The risks faced by non-commercial partners of sex workers have also been studied. An examination of CSW in a Mexican border town with high migratory traffic found that unprotected sex was often common in their personal relationships, too [29]. The literature relating to CSW and travel showed that multiple parties are implicated in commercial sex networks, and the behaviour of any one individual in these networks has implications for many others. Table 3 below summarises these findings.

**Table 3:** Summary of commercial sex worker studies

Study	Destination	Principal Findings
De et al. [24]	Central African Republic	Poor use of condoms by ‘Pupulenge’ with clients
Johnson et al. [13]	Jamaica	Poor condom usage among male sex workers Multiple partners frequent among male sex workers
Avery et al. [25]	Singapore	Lower rates of condom usage when patronising CSW overseas
Lau et al. [26]	Hong Kong	Lower rates of condom usage when visiting CSW overseas
Hsieh et al. [27]	–	Proposal that spread of STI could be greater via networks created by the clientele of CSW
Syvertsen et al. [29]	Mexico	Unprotected sex also prevalent in the personal relationships of CSW

*CSW* commercial sex workers; *STI* sexually transmitted infections

### Sexually transmitted infections

The association of sex tourism and casual sex during travel with the spread of novel STIs has long been recognised. It has been suggested that Columbus’ sailors were responsible for the epidemic of venereal syphilis in Europe in the late fifteenth century following sexual relations with local Haitian women [2], while the link between travel and the spread of novel STIs was also established in Thailand in the 1980s [30], and Trinidad and Tobago in 2012 [31]. Travellers are also thought to be implicated in the reintroduction of syphilis and lymphogranuloma venereum to parts of North America and Europe [25]. The risk factors for traveller acquisition of STIs include longer duration of stay, travel to lower income countries, being single, substance abuse, being male, repeat visits to the same area, and a previous history of multiple partners or STIs [32, 33]. Crawford et al. identified being female, having a history of fewer sexual partners, and having received pre-travel health advice and vaccinations as being associated with a lower risk of contracting STIs among expatriates and travellers [32].

While prevalence rates for STIs among CSW vary, rates as high as 88% in Nairobi and 44% in Bangkok have been reported [34]. In addition to this, high rates of curable STI prevail worldwide, ranging from 5 to 65% in Africa, 20.9% in Brazil and 0–13.6% in Asia [10]. These findings put sex tourists at very high risk for STIs on a global scale. A diverse range of STIs has been recorded in travellers returning from tropical countries [35], from frequent detection of genital herpes in sailors returning to China [36], to the suggested “new” STI *Tinea genitalis*, found in several individuals with a recent travel sex history in Southeast Asia [37]. While this type of dermatophyte infection is not primarily an STI, the sudden rise in cases associated with it over a short period highlights how vulnerable travellers are to organisms transferable through intimate contact during travel.

A study examining all cases of gonorrhoea contracted by people living in Nordic nations between 2008 and 2013 showed that 25.5% of all cases were associated with travel [14]. The rates of travel-associated gonorrhoea increased from year to year and, while the majority of cases involved men, the number of affected women increased from year to year. Among the regions visited, the majority of Nordic travel-associated cases of gonorrhoea were associated with travel to Asia (between December and July) and Europe (from August to November), a third of cases were associated with travel to Thailand, and travel to Thailand, Philippines and Spain accounted for almost half of all travel-related cases. These data imply that specific regions can be considered hotspots for contraction of STIs during travel.

Another important consideration is the acquisition and spread of antimicrobial resistant (AMR) STIs. In recent years, the rise in AMR involving *Haemophilus ducreyi* has been documented worldwide [10]. Similarly, beta-lactamase producing strains of *Neisseria gonorrhoeae* have been detected in Africa, the Caribbean and Asia. In isolates of *N. gonorrhoeae* from Africa and Southeast Asia, penicillin resistance has been reported in as many as 50% of isolates. Baker et al. also noted the worldwide spread of azithromycin-resistant shigellosis through sexual transmission, from high prevalence regions in Africa and Asia, to lower prevalence nations [38]. The documented increase in AMR STIs puts travellers engaging in sexual behaviour at high risk of treatment-resistant infection.

Current efforts to advise and change traveller behaviours have been shown to be of limited effectiveness. A study of different efforts to curtail travellers’ risk behaviour showed that providing brief interventions on sexual health during consults for travellers proved minimally more effective than just distributing condoms or not providing additional advice [39]. This trial showed that the methods employed still resulted in low levels of condom usage. In a study by Croughs et al., extensive motivational training was shown to reduce sexual risk behaviour, and it was also found that written materials on STIs were more effective than having travel health practitioners discuss STI prevention with travellers [40]. A change in strategy appears necessary to combat the risk-taking behaviours of travellers, especially given the reported difficulty of reaching target audiences [41].

This is an important area that warrants further research, given poor recorded levels of condom usage in travellers. A meta-analysis of literature on this subject found that the pooled prevalence of unprotected intercourse among travellers who had sex overseas was 49.4% [42]. Similar results have been shown among sexually active backpackers visiting Ko Tao and Ko Phangan in Thailand, with a third of subjects reporting inconsistent condom use. An online cross-sectional study of travellers was conducted in 2014 [15], and among the sexually active population 59.7% reported inconsistent condom use. A study of condom usage among Swedish travellers revealed flawed reasoning for decisions around condom usage, such as length of familiarity with partner, the country visited, and asking if their partner had an STI [43]. This same study also revealed that some travellers succumbed to peer pressure, were more willing to let their partner make the decision, and had a fear of being seen as promiscuous (among heterosexual women) or a fear of ‘ruining the moment’ (among heterosexual men), leading to reduced condom usage. Other factors associated with reduced usage were the belief that foreign condoms were of poorer quality [34], spontaneous sexual encounters or embarrassment at purchasing condoms [43], substance use [15, 32, 43], and travel to Latin America or the Caribbean [15]. An examination of male sex tourists to Thailand also revealed that unprotected sex was seen as more masculine and enjoyable, and there was a general misconception among male sex tourists that unprotected heterosexual intercourse was a low risk activity [44]. This same study also showed that male heterosexual sex tourists were aware of risks, but due to their own personal or peer experiences being at variance with the warnings they received regarding risky sexual behaviour, they were more likely to engage in unprotected sex with CSW. The low rates of condom usage put sexually active travellers at an obvious risk for contraction of STIs.

It is accepted that contracting an STI increases the risk of HIV transmission, and vice versa [45]. A Geosentinel analysis from 2013 indicated that, out of a sample of 64,335 travellers, 117 returned home with acute symptoms of HIV transmission [46]. In addition, links between clusters of HIV acquisition in Belize, Mexico, Guatemala and Honduras have been found. This finding highlighted the role migration and travel play in the transmission of HIV within Central America. This study also found half of Honduran woman sampled with HIV belonged to viral clusters that were linked to international clusters. Memish and Osoba also noted in their paper on STIs and travel that travellers to Sub-Saharan Africa, Southeast Asia and India were most likely to acquire HIV from unprotected sexual encounters [2]. The voluminous literature relating to STIs and travel indicates that this is an area of key importance to the travel medicine practitioner. While the effectiveness to date of interventions in altering risk behaviours in travellers has been questionable, it is clear that travellers require better access to effective methods of preventing HIV, such as pre-exposure prophylaxis (PrEP), and better education on HIV prevention.

### The LGBTQ+ community and travel sex behaviour

A meta-analysis published in 2018 revealed that gay, bisexual and other men who have sex with men (MSM) travellers were 3 times more likely to have casual sex while travelling [5]. Travel or migration may allow members of the LGBTQ+ community to escape from societal pressures they face in their home countries and explore their sexuality [17]. MSM are also more likely than heterosexual men to have multiple partners during their travels. MSM have also shown to be at least twice as likely to pay for sex compared to heterosexual men [10]. A report on MSM travellers in the United States also found that 19.4% of those surveyed reported that having sex with a new partner was one of their main goals while on vacation [18]. Further studies in the US on MSM travellers to Key West, a popular destination for LGBTQ+ travellers in Florida, found that of the sexually active participants, 34% had new partners, and 59% had unprotected anal intercourse (UAI) [19]. Among Swedish MSM travellers, 13.5% reported UAI during their overseas travels, the majority of whom met a new partner abroad [20]. Additional studies in China involving MSM found that 5% identified as sex tourists, a third of this group identified the purchase of sex as a primary reason for travel, and another third had UAI while travelling [21].

While limited research exists on other categories of travellers within the LGBTQ+ community, one paper on transgender women in Bangladesh revealed that those who crossed international borders had a greater number of transactional sex partners and reduced use of condoms [22]. Across all of these studies, regular associations between travel and drug and alcohol use, transactional sex, group sex, a history of STIs and a greater number of past partners were reported [18–23].

Another interesting area of development in LGBTQ+ international travel trends is the resurgence of circuit parties [47]. These parties involve weekend-long social activities and dance events. Party-goers were found more likely to have a greater number of partners in the previous 6 months, greater use of recreational drugs, more likely to seek transactional sex, and more likely to report a personal history of STI and UAI. A common finding with these parties was attendees travelling from low HIV prevalence countries to high prevalence countries. This finding was replicated among Chinese MSM travellers [21]. These social events are commonly associated with the use of drugs which heighten sexual arousal, an activity referred to as ‘chemsex’.

Networks of MSM travellers have also been described around the world. A group of MSM referred to as “Geoflexibles” was identified by Gesink et al. in 2018 [48]. The authors described a group of men who were willing to travel for sex, and who were less particular about where they had sex. Gesink proposed that these travellers could act as a bridge between MSM in Toronto and, although his study did not specifically mention international travel, it is certainly applicable in the travel context. Networks of MSM implicated in the transmission of STIs and HIV have been suggested in the literature. Persson et al. suggested the presence of a network in Sweden with a high prevalence of STI/HIV [20], and an examination of HIV clusters in Central America found that half of the people living with HIV were MSM, with serotypes closely related to international clusters [49]. The suggestion of international MSM networks and travel playing a role in the dispersion of STI/HIV was reinforced by Takebe et al. in 2014 [50]. Their research revealed the worldwide dispersal of the JP.MSM.B1 subtype of HIV, and confirmed the interactions of HIV epidemics between Japan, China and the rest of the world. These networks have also been implicated in Shigella transmission in San Francisco [51], in addition to an outbreak of Hepatitis A in Northern Italy [52].

These findings have implications for LGBTQ+ travellers who engage in sexual behaviour while abroad. Mathematical modelling of LGBTQ+ tourists to Key West estimated that 1 in 196.5 MSM who engage in risk behaviour will acquire HIV [19], roughly equating to 200 new infections per 100,000 tourists, a number which could drop to as low as 45 with consistent condom use. In 77% of sexual interactions in this study, HIV serostatus was not discussed. Studies about MSM travellers in San Francisco showed that, among those who engaged in casual sex, there was a decreased probability of HIV serodisclosure when communication was an issue owing to language barriers [53]. A follow up study was conducted on the health-seeking behaviour of MSM travellers, revealing that a quarter of those surveyed had not received the Hepatitis B virus vaccine, and of the men living with HIV, a third had not been vaccinated [54].

Another facet of the intersection between sex tourism and HIV transmission that warrants attention is the relatively new phenomenon of “holiday pre-exposure prophylaxis” (PrEP) for HIV. With PrEP being a relatively new phenomenon, limited literature exists on the subject in relation to travel, but interviews conducted by Underhill et al. suggest that MSM travellers regard themselves as at greater risk for HIV while travelling and are more willing to take PrEP [55]. However, travel has also been associated with disruption in PrEP regimens due to inconvenience [56, 57], so the role it plays in sex tourism warrants further research.

Travel for the purposes of sexual exploration and casual sex among MSM presents a challenge to travel medicine practitioners. Analysis of Swedish MSM travellers in 2015 revealed that there was little HIV or STI prevention information received in Sweden or abroad [58]. In addition, only 3% of the surveyed population sought out this information before travelling. A further investigation of the knowledge, attitudes and practices of MSM travellers is required to plan successful interventions in this population of international travellers. More research on how sex tourism is experienced by women and gender diverse people within the LGBTQ+ community is also warranted.

### The effects of alcohol and drugs on sex tourism

Drugs and alcohol play a major role as risk factors for and cofactors in casual sexual behaviour while abroad. A study of British summer workers in Ibiza found that almost all those surveyed drank alcohol, while 85.3% used drugs during their stay, a high proportion of whom used drugs that they had never tried before [59]. This study found that the odds of having sex increased with the use of amphetamines or higher frequency of drinking, while the odds of having multiple partners increased with greater frequency of drinking. Unprotected sex was also found to be more likely when alcohol was involved.

Extensive analysis of American students on Spring Break has also been conducted to analyse the role alcohol plays in high risk behaviour during this period. Patrick et al. found that a greater proportion of students drank alcohol before having sex or making risky sexual decisions [60]. This finding was particularly prevalent among students who travelled abroad. Another study of Spring Break students found that risky behaviours such as unprotected sex or multiple partners were cumulative [61], such that engaging in one activity increased risk for the other. Almost half of the students in this study reported binge drinking before sex. The role alcohol and drugs play in exposing travellers to risky sexual behaviour is clear, but this appears to be poorly appreciated by the traveller. Travel health practitioners must emphasise the risks travellers expose themselves to when misusing alcohol and drugs.

### Sexual assault and violence in travellers

A cross-sectional survey on travellers returning from Mediterranean resorts reported that 1.5% were subject to non-consensual sex during their travels, with gay and bisexual males reporting higher levels [62]. In this same report, 8.6% of respondents experienced some form of sexual harassment, with females and gay/bisexual males more frequently reporting this. Another finding was that being a gay/bisexual male, using marijuana, and patronising bars where there were opportunities for sex were factors associated with being subject to non-consensual sex. A similar study on the harassment of tourists in Barbados found between 7 and 12% of tourists reported sexual harassment, depending on their country of origin [63]. Kennedy and Flaherty also asserted that up to 4% of Irish citizens reporting sexual violence experience it while travelling [64]. A review from Canada of all reported sexual assault cases associated with mass gathering events found a significant association between being overseas and being sexually assaulted at such an event [65]. Table 4 outlines the pre-travel health advice which should be available to travellers who may engage in sex tourism.

**Table 4:** Pre-travel health recommendations

Clinicians should ask travellers if they intend on engaging in sexual intercourse while overseas.	
Recommend traveller to carry condoms and other forms of protection if they intend to engage in intercourse overseas.	
Provide travellers with written materials on STI prevention, in addition to providing a verbal intervention.	
Offer condoms and sexual lubricant to the patient, if possible.	
Assess travellers’ suitability for HIV PrEP.	

*STI* sexually transmitted infection; *HIV* human immunodeficiency virus; *PrEP* pre-exposure prophylaxis

### Child sex tourism

Klain described two main types of child sex tourist, the “elective sex tourist” who travels for leisure or business and makes unplanned use of child sex workers when given the opportunity, and the “core sex tourist”, the purpose of whose trip is solely to engage in sexual contact with a child [66]. A study of German tourists conducted in 2017 found that 0.4% reported being child sex tourists [67]. This same study found that these individuals usually had personal experiences of abuse, paedophilic and antisocial behaviours. With an estimated 1.2 million children trafficked worldwide annually [67], more research is urgently needed on this topic.

### The effects of wealth and mobility on sex tourism

Aggleton et al. describe in their paper a specific group of travellers, “mobile men with money” [68]. These men come from diverse backgrounds and various employments, but share two common features, high spending power and high mobility. In the paper, these men were said to frequently use their high spending power and resources to engage in casual and transactional sex encounters. This group of men was found to be at high risk for HIV. The paper proposed that these men lacked social support and were frequently influenced by the behaviour of their peers. While further literature on this sub-group is lacking, travel to lower income countries and the resulting increase in spending power for the traveller have been documented as risk factors for acquisition of STI/HIV [32]. This would suggest that wealth inequality may have a role in influencing risk behaviours in certain individuals.

### Impact of sex tourism on host communities

While a detailed consideration of the impact of sex tourists on sex tourism destinations is beyond the scope of the current work, some key issues are worthy of discussion. Local cultural attitudes towards sex tourism are complex and are influenced by harsh economic conditions, where impoverished families may find themselves with few options for survival and have to resort to sending their children to urban centres visited by sex tourists. There may be an expectation in some cultures that children will share the family’s financial burden. Remittances from a family member engaged in the sex tourism industry may be vital to enable families to improve their quality of life.

Child sex tourism produces a detrimental impact on the children’s capacity to achieve their goals within the education system. Sex tourism may reinforce traditional colonial attitudes towards race and gender, which serve to deepen existing socioeconomic inequalities. Local communities are often reluctant to intervene in cases of child sexual exploitation, given the complex underlying economic precipitants and the greater level of public acceptability of prostitution in some countries. Such attitudes render children far more vulnerable to being absorbed by the adult sex trade and becoming sexually exploited by sex tourists, who may use the anonymity afforded by the dark web as a global networking tool to share information with other sex tourists.

The COVID-19 pandemic has led to school closures and a higher risk of contact between children and online sexual predators. It has isolated victims of child trafficking and sex tourism from available support structures and jeopardised their usual escape routes. The reported 30% increase in consumption of online child pornography during recent periods of pandemic lockdown in Europe, for example, have further increased the demand for child exploitation [69]. The current restrictions on international travel will undoubtedly influence sex tourism patterns worldwide, leading to greater degrees of domestic child abuse and online sexual exploitation. Further research may shed a light on this and other COVID-related secondary effects on the sex tourism industry.

## Discussion

### Future considerations in sex tourism

While the world prepares for a cautious return to routine international travel in a future post-COVID era [70], we may ponder what constitutes a traveller or a tourist in the modern era. Opperman proposed the idea of a ‘cyberspace tourist’ in his paper on sex tourism [71]. While we have not found any further literature on this subject, is a person who sits at a computer in his/her home and pays for a voyeuristic virtual reality experience involving a foreigner thousands of miles away a cyber-sex tourist? With the rapid advancements in technology in recent years, we may contemplate whether people even need to leave their home to “travel”. It is conceivable that future sexual experiences will mirror these changes in travel patterns. With PrEP being a recent development, the role it plays in protecting travellers exposed to HIV overseas remains to be seen. This is a potential area of research activity as it becomes established as a mainstay preventive option. Possible areas of unmet need in sex tourism research are presented in Table 5.

**Table 5:** Sex tourism research priorities

Redefining “sex tourism”	
Investigating the role sex tourism websites play in promoting and encouraging sex tourism	
Developing strategies for supporting the physical and mental health of sex workers involved in sex tourism	
Designing strategies for ensuring safe sexual behaviour while travelling	
Examining the use of PrEP in international travellers	

*PrEP* pre-exposure prophylaxis

### Limitations of current review

Strengths of our review include its multidisciplinary authorship, its broad coverage of diverse facets of sex tourism, and the focus on the most recent literature on the subject. Limitations of our approach include its restriction to articles published in the English language and the use of a single medical literature database. Accessing literature on sex tourism from Latin America and the Caribbean, using the Latin American and Caribbean Health Sciences Literature virtual library, for example, may have provided deeper insights into the impact of sex tourism on host communities. It is reasonable to assume that relevant literature on sex tourism resides in the social sciences literature such as the Social Sciences Citation Index of the Web of Science. Future reviews on this topic should also consult an appropriate social sciences database and refer to relevant material from the anthropological literature.

## Conclusions

In our review of the literature associated with sex and travel, it was clear that the same set of risk behaviours and consequences applied to diverse groups. We recommend that more research be conducted into novel and effective interventions for modifying these high-risk behaviours. Travellers should be informed of the increased risks of STI before they travel. They should be aware of the prevalence of STIs in the area they plan to visit, and the risks associated with their sexual practices when they travel, including engaging with commercial sex workers, practising chemsex, engaging in unprotected sexual intercourse, and becoming the victim of sexual violence. They should also be informed about how to access appropriate medical care overseas and as returned travellers, should they require it.

## Data Availability

All material referenced in the preparation of this work are available from the corresponding author.
